# Knowledge about the administration and regulation of high alert medications among nurses in Palestine: a cross-sectional study

**DOI:** 10.1186/s12912-019-0336-0

**Published:** 2019-03-20

**Authors:** Sa’ed H. Zyoud, Samar M. Khaled, Baraa M. Kawasmi, Ahed M. Habeba, Ayat T. Hamadneh, Hanan H. Anabosi, Asma’a Bani Fadel, Waleed M. Sweileh, Rahmat Awang, Samah W. Al-Jabi

**Affiliations:** 1 Poison Control and Drug Information Center (PCDIC), College of Medicine and Health Sciences, An-Najah National University, Nablus, 44839 Palestine; 2 Division of Clinical and Community Pharmacy, College of Medicine and Health Sciences, An-Najah National University, Nablus, 44839 Palestine; 3 PharmD program, College of Medicine and Health Sciences, An-Najah National University, Nablus, 44839 Palestine; 4 Department of Pharmacology and Toxicology, College of Medicine and Health Sciences, An-Najah National University, Nablus, 44839 Palestine; 5 WHO Collaborating Centre for Drug Information, National Poison Centre, Universiti Sains Malaysia (USM), 11800 Penang, Malaysia

**Keywords:** Nurses’ knowledge, High alert medications, Medication errors

## Abstract

**Background:**

Medication errors (MEs) are unintended failures in the drug treatment process that can occur during prescription, dispensing, storing, preparation or administration of medications. High alert medications (HAMs) are defined as those medications that bear the highest risk of causing significant patient harm when used incorrectly, either due to their serious adverse events or to a narrow therapeutic window. Nurses are responsible for administration of HAMs; incorrect administration can have a significant clinical outcome. This study aimed to assess the level of knowledge of HAMs among nurses in government hospitals in West Bank, Palestine.

**Methods:**

A cross-sectional study was conducted in 2015, in West Bank, Palestine. Data were collected via a face to face interview questionnaire, which was taken from a previous study. Data were collected by convenient sampling. The questionnaire consisted of four parts: demographic characteristics of the nurses, drug administration knowledge (10 true-false questions), drug regulation knowledge (10 true-false questions), and self-evaluation.

**Results:**

A total of 280 nurses participated in the study; these nurses were working in the emergency room (ER), intensive care unit (ICU), paediatric or medical ward. The response rate was 93%. Nurses were found to have insufficient knowledge about HAMs; 67.1% of participants had a score of less than 70%, with a mean total score of 59.9 ± 15.1. Factors associated with sufficient knowledge among nurses were HAMs training and ICU training, both with *p*-values of 0.002. Nurses with a master degree, those working in the ICU ward, head nurses, and male nurses were the most knowledgeable groups, with a p-values < 0.001. 81.8% of respondents hoped to obtain additional training. The leading obstacles reported were inconsistent opinions between doctors and nurses (37.9%), and no established standard operating procedure for HAMs (37.1%).

**Conclusions:**

Lack of knowledge was one of the obstacles that nurses encountered during administration of HAMs which might result in MEs. Nurses reported that they would like to have additional training to update their pharmacology knowledge. Nurses could benefit from additional continuing education and training programs.

**Electronic supplementary material:**

The online version of this article (10.1186/s12912-019-0336-0) contains supplementary material, which is available to authorized users.

## Background

Medication safety during administration is a major concern at a global level, and is related to safety and quality of patient care [1–3]. Medication errors (MEs) are unintended failures in the drug treatment process that can occur during prescription, dispensing, storing, preparation and administration of medications. MEs are one of the major concerns of nursing professionals internationally [4]. High alert medications (HAMs) are considered to be one of the most medications associated with a high risk of serious harm if administered improperly, and are responsible for the majority of harmful errors [5–13]. [5–13]. Nurses are responsible for administration of HAMs; incorrect administration can have a significant clinical outcome, and at times can be fatal. A considerable amount of literature has been published on the pharmacology knowledge of nurses [5–9]. Overall, these studies highlight the need for more research to evaluate nurses’ knowledge of pharmacology for the purpose of drug administration [5–10].

The American Pharmaceutical Association classifies HAMs according to the following list of categories: chemotherapeutic agents (oral and parenteral), cardiovascular medications (e.g., adrenergic drugs), narcotics (e.g., morphine or fentanyl), anticoagulants (e.g., warfarin and heparin), neuromuscular blocking agents (e.g., rocuronium or succinylcholine), benzodiazepines (e.g., midazolam) and electrolytes (e.g., 15% potassium chloride (KCl)) [11]. HAMs are commonly used in the emergency room (ER), intensive care unit (ICU), paediatric ward and medical ward. Because HAMs are used in emergency situations, they bear a heightened risk of causing patient harm when used incorrectly. Some HAMs have a narrow therapeutic index, e.g., warfarin, which when used improperly, rapidly causes the undesirable side effect of bleeding. Further, well-known chemotherapeutic agents, such as vincristine, require special handling, and should be administered according to the manufacturer’s recommendations, “FOR INTRAVENOUS (IV) USE ONLY”. If administered by another route of administration, e.g., intrathecal, it will cause significant patient harm, and even death. Moreover, most nurses fall into error while calculating chemotherapeutic doses. For example, they may use patient body weight (BW), when most chemotherapeutic doses are determined based on body surface area (BSA). Further, one of the most common factors contributing of patient harm is fast infusion rate of HAMs, e.g., fast IV bolus push of KCL and insulin.

The nurse has many tasks to do in Palestine, and likewise, in most countries around the world, to reach correct and safe administration. These tasks include preparing medications, administrating medications, reporting adverse drug reactions, monitoring the effectiveness of treatment, and counselling patients about their medications. Several studies have been conducted among nurses in Palestine to investigate their knowledge about many topics [12–16], but none of these studies were about HAMs, which are the most dangerous and critical medications. Inadequate nurse education, and inadequate experience in administration, storage and dose calculation, increases the risk of MEs. To the best of our knowledge, there is no work investigating the knowledge of HAMs among nurses in Palestine. There is a lack of studies on this topic both in Palestine and in the surrounding countries, and it is considered one of the factors that make our health services and organizations poor and incompetent. In this study, we tried to determine if nurses had adequate pharmacology knowledge of HAMs administration and regulation in West Bank, Palestine. This study will try to uncover these weaknesses and gaps in our health system, and will focus on finding optimal solutions, which is considered an urgent need. Further, this study will assist in the design of new strategies for teaching, training and practice. Nurses need up to date medication education so they are able to provide optimal care to patients, and remain safe in their professional field.

## Methods

### Study design

This study had a cross-sectional design. Nurses from multi-specialty hospitals in Palestine were surveyed regarding their knowledge of HAMs.

### Study area

Nursing staff in the government hospitals were our target participants because HAMs are usually used in hospitals. We included four wards: the ER, ICU, paediatric and medical ward. This study focused on governmental hospitals in West Bank, Palestine; we chose governmental hospitals because they have a heavy work load and contain the largest number of nurses. The main hospitals from the six different districts in West Bank were included in this study: Beit-Jala hospital in Bethlehem, Alia hospital in Hebron, Palestine Medical Complex (PMC) in Ramallah, Rafedia and Al-Watani hospitals in Nablus, Thabet Thabet hospital in Tulkarm and Jenin hospital in Jenin [17].

### Population

Both males and females were included in this study, spanning a wide age range. The study focused on the aforementioned wards (ER, ICU, paediatric and medical) because they carry the highest risk of MEs. Nurses on the selected medical wards use HAMs a lot because patients tend to have multiple complications, and thus need complex medication regimens, which may lead to MEs.

### Sample size

Based on data from the Palestinian Health Information Centre in 2014 [17], there are 1566 nurses working in the government hospitals in West Bank, Palestine. Overall, 1225 nurses working in the hospitals were included in the current study. Based on information obtained from the head nurse of each department, approximately 432 nurses working in the ER, ICU, paediatric and medical wards at each hospital were included. The sample size needed for this study was calculated using the Raosoft sample size calculator [18] to achieve a confidence level of 95% and a margin of error of 5%. The estimated sample size was 204 nurses. The target sample size was increased to 280 nurses to be statistically adequate.

### Sampling technique

Data collection occurred from the beginning of June 2015 to the end of July 2015. Data was collected by convenient sampling. The sample was taken from six districts and clustered into three areas: north (Nablus, Tulkarm and Jenin), middle (Ramallah) and south (Bethlehem and Hebron). This was so that the sample could be representative.

The head nurse of each department was visited, and the investigator explained the aims and objectives of the study. The number of working nurses in each department, and the times at which nurses change shifts, was provided by the head nurse. Each hospital was visited approximately seven times, and each ward was visited at least four times, in order to cover all nurses working in different shifts (A, B and C). The ward was visited at a time when the shifts were changing, in order to access nurses from two different shifts.

After receiving permission from the head nurse, nurses were individually interviewed at their working sites, face to face. The data collection form was administered, making sure that each nurse understood the objectives of the study and how to fill it in. The interviewer was able to explain any misunderstandings during the data collection process.

### Inclusion and exclusion criteria

The inclusion criteria were as follows: Palestinian nationality, licensed nurse in Palestinian ministry of health, at least diploma certification, or higher degree, and works in ER, ICU, paediatric or medical ward. The Exclusion criteria were as follows: Refused to participate in the study, intern nurse or nursing school student, and works in private or teaching hospitals.

### Data collection form

A face-to-face interview data collection tool was developed in Arabic language. The study instrument was obtained from a study in Taiwan, titled ‘Nurses’ knowledge of HAMs: Instrument development and validation’ [19]. The data collection form consisted of four parts (See Additional file 1).

#### Part a: demographics

This section asked about demographic information such as age, gender, position, year of graduation, years of experience and ward where currently working. We classified age into four different categories: less than or equal to 25 years, 25 to 30 years, 30 to 35 years, and more than 35 years. Graduated nurses were eligible for participation, regardless of years of experience; however, years of experience was classified into four categories: less than or equal to 2 years, 2 to 5 years, 5 to 10 years, and more than 10 years. We also included nurses with various education degrees (diploma, bachelor and master) [19].

#### Part B: HAMs administration knowledge

This section contained 10 questions, each with three response options; ‘true’, ‘false’ and ‘don’t know’. This section asked about HAMs administration. The questions focused on drug dosage and delivery routes, for example, 15% KCl, epinephrine, 10% calcium chloride (CaCl_2)_ and 5% sodium chloride (NaCl) are not administered by IV bolus injection; the dosage expression for insulin is units, so it is not administered by mL syringe. Total score for this section was 10 points, with one point given for each correct answer, and zero for incorrect and ‘don’t know’ answers.

#### Part C: HAMs regulation knowledge

This section contained 10 questions with the response options ‘true’, ‘false’ and ‘don’t know’. These questions were focused on HAMs regulation, including storage, regulation and writing of HAMs. For example, insulin and heparin should be stored in individual storage containers to avoid mistakes; fentanyl patches should be considered as regulated narcotics; muscle relaxants and 15% KCl should be stored in a locked and safe place; vials, teaspoons or “U” are not the correct ways to express dosages. Total score for this section was 10 points; one point was given for each correct answer and zero for incorrect and ‘don’t know’ answers.

Each correct answer in part B and C (total of 20 questions) was given five points, with a total score of 100; incorrect or don’t know answers were given a score of zero. Knowledge scores were classified as good knowledge ≥70%, or poor knowledge < 70%.

#### Part D: self-evaluation

This section was designed to evaluate nurses’ knowledge; the questions were divided into three parts:

- Obstacles nurses encounter when administering HAMs: 14 obstacles as multiple choice questions; participant can choose more than one option.
- Nurses’ knowledge level: consisting of five levels (sufficient, relatively sufficient, fair, insufficient and extremely insufficient).
- Training needs: these questions had three response options, ‘need’, ‘no need’ and ‘no comment’.

Permission to use the instrument and to translate it to Arabic was obtained from the author. The HAMs instrument was translated by a standard “forward–backward” procedure following recommended international guidelines [20–22]. The questionnaire was then tested for face and content validity with three experts in the fields of public health research, medical education, and research design to assess the organization, meaning of terms, completeness, logical sequence of the statements, and the accuracy. Before starting data collection, a pilot study was undertaken to ensure that the data collection form was applicable and convenient. Twenty nurses were surveyed from different districts and departments. Results from the pilot testing were not included in the final analysis of the data. Nurses were asked to provide their opinions and comments about the instrument. Most of them commented on question nine part B (taken fentanyl skin patch as regulated narcotic), indicating that they did not know that there is a fentanyl patch.

### Ethical approval

Approval to conduct this research study was granted by An-Najah National University Institutional Review Board, and the local health authorities from Ministry of Health (MOH), before initiation of this study. Furthermore, permission to collect data from the nursing staff was sought (and received) from the head nurse of every ward. Verbal consent was obtained from the nurses before starting data collection.

### Statistical analysis

Categorical variables are presented as frequencies with percentages. Continuous data are presented as means with SDs, or medians with lower–upper quartiles. Continuous data was tested for normality using the Kolmogorov–Smirnov test. Nonparametric continuous data was compared between two groups using the Mann-Whitney U test, and between more than two groups using the Kruskal-Wallis test. Furthermore, categorical data was compared between groups using the chi-square or the Fisher exact test, as appropriate. Pearson’s correlation coefficient was calculated to examine a possible correlation between continuous variables (administration knowledge score and regulation knowledge score). All statistical analyses were carried out with the use of SPSS software (version 16.0). A *p* value < 0.05 was considered statistically significant.

## Results

In total, 301 nurses who were working in the included wards (ER, ICU, paediatric and medical) in seven governmental hospitals were approached to participate. Out of the 301 nurses, 280 nurses completed the questionnaire, with a response rate of 93%.

### Demographic characteristics

The demographic characteristics of the nurses are represented in Table 1. One hundred and fifty three (54.6%) nurses were males; the majority of nurses (121; 43.2%) were between the ages of 25-30 years. With regard to the district where the nurses were working, the majority of the nurses came from Nablus (74; 26.4%).

**Table 1:** Demographic characteristic of survey respondents among the nursing staff

Variable	Frequency (%) *N* = 280
Gender	
Male	153 (54.6)
Female	127 (45.4)
Age (year)	
≤25	37 (13.2)
> 25–30	121(43.2)
> 30–35	64 (22.9)
> 35	58 (20.7)
District	
Hebron	44 (15.7)
Bethlehem	38 (13.6)
Ramallah	58 (20.7)
Nablus	74 (26.4)
Tulkarm	26 (9.3)
Jenin	40 (14.3)
Education	
Diploma	96 (34.3)
Bachelor	161 (57.5)
Master	23 (8.2)
Position	
Qualified Nurse^a^	96 (34.3)
Legal Nurse^b^	170 (60.7)
Head Nurse	14 (5.0)
Wards	
ICU	71(25.4)
ER	62 (22.1)
Pediatrics	75 (26.8)
Medical	72 (25.7)
Ward experience (year)	
< 2	95 (33.9)
> 2–5	89 (31.8)
> 5–10	62 (22.1)
> 10	34 (12.1)
Total experience (year)	
< 2	25 (8.9)
> 2–5	69 (24.6)
> 5–10	106 (37.9)
> 10	80 (28.6)
ICU training	
Yes	200 (71.4)
No	80 (28.6)
ER training	
Yes	234 (83.6)
No	46 (16.4)
HAMs training	
Yes	252 (90.0)
No	28 (10.0)

*ER* Emergency room, *ICU* Intensive care unit, *HAMs* High-alert medication

^a^Qualified nurse with professional diploma degree in nursing

^b^Legal nurse with bachelor of science degree in nursing as the minimum educational requirement for professional nursing practice

The majority of nurses (161; 57.5%) had a bachelor degree in nursing. Most of the nurses were legal nurses (170; 60.7%). Seventy-one (25.4%) of the nurses included in the study were from the ICU, 62 (22.1%) were from the ER, 75 (26.8%) were from the paediatric ward and 72 (25.7%) were from the medical ward. Ninety-five nurses (33.9%) had less than two years of experience, while the majority of nurses (106; 37.9%) had 5–10 years of total experience. In total, 200 (71.4%) nurses had ICU training, 234 (83.6%) had ER training and 252 (90%) had HAMs training (Table 1).

### HAMs administration knowledge

When asked about proper administration of HAMs, the overall correct response rate was 60.9%; 23.9% were incorrect answers, and 15.2% were ‘don’t know’ answers. In contrast, question number 10, “about whether Port-A route can generally be used for blood withdrawal and drug injection”, received the lowest correct response rate: only 30.7% answered correctly. The highest correct response rate was 83.9%, for the question about whether the dosage expression for insulin injections is ‘cc’ or ‘mL’. Further, 53.6% of nurses had a problem with chemotherapy dosage calculation, whereby the correct dosage calculation for adult chemotherapy is based on BSA, and for children it is based on BW. It was surprising that more than one third of nurses (99, 35.4%) agreed with fast IV infusion of 3% NaCl 500 mL for patients who have low sodium levels, as shown in Table 2.

**Table 2:** Nurses’ knowledge of high alert medications administration

Questions^a^ (section A)	Answers	Correct answer *n* (%)	Incorrect answer *n* (%)	Don’t know the answer *n* (%)	Rank
Fast IV push 1: 1000 epinephrine 1 ampule for patient who has mild allergic reaction	False	178 (63.6)	73 (26.1)	29 (10.4)	5
When an emergency happens, fast IV push 10% CaCl_2_ 10 mL in 1–2 min	False	170 (60.7)	76 (27.1)	34 (12.1)	7
10% Ca gluconate and 10% CaCl_2_ are the same drug and interchangeable	False	192 (68.6)	37 (13.2)	51 (18.2)	3
‘cc’ or ‘mL’ is the dosage expression for insulin injection	False	235 (83.9)	25(8.9)	20 (7.1)	1
For chemotherapy dose calculation, while adult based on BW, children BSA	False	130 (46.4)	65 (23.2)	85 (30.4)	9
When an emergency such as ventricular fibrillation happens, push fast 15% KCl 10 mL into IV	False	215 (76.8)	25 (8.9)	40 (14.3)	2
15% KCl better added to Ringer’s solution for rapid infusion	False	187 (66.8)	51 (18.2)	42 (15.0)	4
Insulin syringe can be replaced by 1 mL syringe	False	173 (61.8)	90 (32.1)	17 (6.1)	6
Fast IV infusion of 3% NaCl 500 mL for patient who has low sodium level	False	139 (49.6)	99 (35.4)	42 (15.0)	8
Port-A route can be used for blood withdrawal and drug injection generally	False	86 (30.7)	128 (45.7)	66 (23.6)	10
Mean		60.9	23.9	15.2	

*KCL*, Potassium chloride, *Ca* Calcium, *NaCl* Sodium chloride, *CaCl*_*2*_ Calcium chloride, *IV* Intravenous, *BW* Body weight, *BSA* Body surface area

^a^These questions were adapted from Hsaio et al. [19] with permission from the principal investigator

### HAMs regulation knowledge

This section had a correct response rate of 58.9% and an incorrect response rate of 32.1%; 9.02% were ‘don’t know’ responses. The highest correct response rate was 89.3% for the question asking about the use of distinctive labelling on look-alike drugs. It was surprising that more than half of nurses (148, 52.9%) used “U” instead of unit for dose expression. Further, as shown in Tables 3, 43.2% of nurses believed that potassium cannot be administered orally, instead of the IV route, if the patient can tolerate it.

**Table 3:** Nurses’ knowledge of high alert medications regulation

Questions^a^ (Section B)	Answers	Correct answer *n* (%)	Incorrect answer *n* (%)	Don’t know the answer *n* (%)	Rank
Use ‘Amp’ or ‘Vial’ for dose expression instead of ‘mg’ or ‘gm’	False	223 (79.6)	51 (18.2)	6 (2.1)	2
Use distinctive labeling on look-alike drugs	True	250 (89.3)	19 (6.8)	11 (3.9)	1
Use ‘U’ instead of ‘unit’ for dose expression	False	123 (43.9)	148 (52.9)	9 (3.2)	10
For convenience, heparin and insulin should be stored together in the refrigerator	False	182 (65)	85 (30.4)	13 (4.6)	3
Each drug better have multiple concentrations for nurse to choose	False	138 (49.3)	122 (43.6)	20 (7.1)	6
If patient can tolerate, potassium can be administered orally instead of IV route	True	128 (45.7)	121 (43.2)	31 (11.1)	9
15% KCl is frequently used, so it should be easily and freely accessed by nurses	False	173 (61.8)	90 (32.1)	17 (6.1)	4
For pediatric dose, use teaspoon for dose expression	False	130 (46.4)	134 (47.9)	16 (5.7)	8
Taken fentanyl skin patch as regulated narcotic	True	136 (48.6)	68 (24.3)	76 (27.1)	7
If a ward stores Atracurium for tracheal intubation, the drug should be stored with other drugs and easily accessed by nurses	False	166 (59.3)	60 (21.4)	54 (19.3)	5
Mean		58.9	32.1	9.0	

*Amp* Ampoule, *IV* Intravenous, *KCl* Potassium chloride

^a^These questions were adapted from Hsaio et al. [19] with permission from the principal investigator

### Self-evaluation

Three primary factors were included in the self-evaluation section:

- Encountered obstacles: each nurse chose an average of four to five obstacles, from the 14 obstacles listed. Inconsistent opinions between doctors and nurses was reported to be the most common obstacle encountered (106, 37.9%), while easy access to HAMs was the least encountered obstacle (33, 11.8%). See Table 4.
- Knowledge level: most respondents (106, 37.9%) considered that they had relatively sufficient knowledge. Only 2.1% of respondents evaluated their knowledge as extremely insufficient. See Table 5.
- Training needs: most of respondents (229, 81.8%) hoped to obtain additional training. See Table 5.

**Table 4:** Obstacles nurses encounter when administering high-alert medications (*n* = 280; multiple responses)

Obstacles	*N*	(%)
Inconsistent opinions between doctor and nurse	106	37.9
No established standard operating procedures for high alert medication	104	37.1
No reference for drug use	98	35.0
Have to accept oral order	75	26.8
Insufficient knowledge	67	23.9
Confused prescription	64	22.9
Unclear dose calculation	64	22.9
Find no suitable person to consult	60	21.4
Mix high-alert medications with other drugs	60	21.4
No rigorous regulations for high-alert medications	59	21.1
Receive uncertain answers from colleagues	51	18.2
Inconsistent opinions between nurses	42	15.0
Easy access to high-alert medications	33	11.8
Other	20	7.1

^a^Many of these obstacles were adapted from Hsaio et al. [19] with permission from the principal investigator

**Table 5:** Self-evaluation knowledge level and training needs for high-alert medications (*n* = 280)

Knowledge level	*N*	(%)
(a) Sufficient	66	23.6
(b) Relatively sufficient	106	37.9
(c) Fair	87	31.1
(d) Insufficient	15	5.7
(e) Extremely insufficient	6	2.1
Total	280	100
Training need		
(a) Need	229	81.8
(b) No comment	33	11.8
(c) No need	18	6.4
Total	280	100

### HAMs knowledge score

The median HAMs knowledge score was 60.0 (interquartile range: 50.0–70.0) and the mean score was 59.9 ± 15.1. As shown in Table 6, significant differences in HAMs knowledge scores were found according to gender, ICU training and HAMs training (Mann-Whitney U test; *p*-value < 0.05), as well as educational level, hospital ward and nurse’s position (Kruskal-Wallis test; *p*-value < 0.05). Male nurses had higher HAMs knowledge scores, and HAMs knowledge score increased as the educational level and nurses’ position increased. Further, HAMs knowledge score was lower for nurses who did not have ICU and HAMs training, compared to those who had. Furthermore, ICU nurses had the highest HAMs knowledge scores.

**Table 6:** Factors associated with nurses’ high-alert medications knowledge score

Variable	Frequency (%) *N* = 280	Knowledge score Median [interquartile range]	Mean ± SD	*P*-value
Gender				
Male	153 (54.6)	65.0[50.0–75.0]	62.5 ± 15.0	0.001^a^
Female	127 (45.4)	55.0[45.0–65.0]	56.9 ± 14.8	
Age (year)				
< 25	37 (13.2)	60.0[45.0–67.0]	57.0 ± 15.2	
> 25–30	121 (43.2)	60.0[50.0–70.0]	58.2 ± 14.5	0.07^b^
> 30–35	64 (22.9)	65.0[51.0–75.0]	63.4 ± 15.5	
> 35	58 (20.7)	65.0[50.0–75.0]	61.5 ± 15.4	
District				
Hebron	44 (15.7)	65.0[45.0–75.0]	60.5 ± 16.5	0.085^b^
Bethlehem	38 (13.6)	60.0[50.0–66.3]	59.1 ± 14.0	
Ramallah	58 (20.7)	65.0[50.0–75.0]	63.4 ± 16.1	
Nablus	74 (26.4)	62.5[45.0–70.0]	60.3 ± 14.1	
Tulkarm	26 (9.3)	55.0[43.8–65.0]	52.3 ± 15.2	
Jenin	40 (14.3)	60.0[50.0–70.0]	59.4 ± 13.9	
Education				
Diploma	96 (34.3)	55.0[45.0–65.0]	55.3 ± 15.4	0.001^b^
Bachelor	161 (57.5)	65.0[50.0–75.0]	62.2 ± 14.5	
Master	23 (8.2)	65.0[50.0–75.0]	63.7 ± 14.5	
Position				
Qualified Nurse ^c^	96 (34.3)	55.0[45.0–65.0]	55.3 ± 15.4	0.001^b^
Legal Nurse ^d^	170 (60.7)	65.0[50.0–75.0]	62.0 ± 14.5	
Head Nurse	14 (5.0)	67.5[53.8–80.0]	66.8 ± 13.8	
Wards				
ICU	71 (25.4)	70.0[55.0–75.0]	65.4 ± 14.4	0.001^b^
ER	62 (22.1)	60.0[45.0–70.0]	58.1 ± 15.6	
Pediatrics	75 (26.8)	55.0[45.0–65.0]	56.4 ± 15.1	
Medical	72 (25.7)	60.0[50.0–70.0]	59.7 ± 14.2	
Ward experience (year)				
< 2	95 (33.9)	60.0[45.0–70.0]	57.9 ± 15.3	0.178^b^
> 2–5	89 (31.8)	60.0[47.5–70.0]	59.1 ± 15.0	
> 5–10	62 (22.1)	65.0[50.0–75.0]	62.9 ± 14.1	
> 10	34 (12.1)	65.0[50.0–75.0]	62.4 ± 16.1	
Total experience (year)				
< 2	25 (8.9)	55.0[42.5–72.5]	54.8 ± 18.7	0.154^b^
> 2–5	69 (24.6)	60.0[45.0–65.0]	57.8 ± 14.1	
> 5–10	106 (37.9)	65.0[50.0–70.0]	60.8 ± 14.6	
> 10	80 (28.6)	65.0[50.0–75.0]	62.3 ± 15.1	
ICU training				
Yes	200 (71.4)	65.0[50.0–75.0]	61.7 ± 15.0	0.002^a^
No	80 (28.6)	55.0[45.0–65.0]	55.5 ± 14.6	
ER training				
Yes	234 (83.6)	60.0[50.0–71.3]	60.6 ± 15.5	0.104^a^
No	46 (16.4)	55.0[45.0–70.0]	56.5 ± 15.2	
HAMs training				
Yes	252 (90.0)	65.0[50.0–75.0]	60.9 ± 14.8	0.002^a^
No	28 (10.0)	50.0[41.3–60.0]	50.9 ± 14.9	
Training need				
Need	229 (81.8)	60.0[47.5–70.0]	59.7 ± 15.1	0.848^b^
No comment	33 (11.8)	60.0[50.0–75.0]	60.9 ± 16.4	
No need	18 (6.4)	60.0[48.8–72.5]	61.1 ± 13.8	

*ER* Emergency room, *ICU* Intensive care unit, *HAMs* High-alert medication

^a^Statistical significance of differences calculated using the Mann–Whitney U test

^b^Statistical significance of differences calculated using the Kruskal–Wallis test

^c^Qualified nurse with professional diploma degree in nursing

^d^Legal nurse with bachelor of science degree in nursing as the minimum educational requirement for professional nursing practice

### HAMs administration knowledge score

The median HAMs administration knowledge score was 6.0 (interquartile range: 5.0–8.0), and the mean score was 6.1 ± 2.0. As shown in Table 7, significant differences in HAMs administration knowledge scores were found according to gender and HAMs training (Mann-Whitney U test; *p*-value < 0.05), as well as age, and hospital ward (Kruskal-Wallis test; p-value < 0.05). Nurses aged more than 35 years had higher HAMs administration knowledge scores than those aged less than 35 years. Male nurses also had higher HAMs knowledge scores than females, and HAMs administration knowledge score was higher among nurses who had HAMs training compared to those who did not. ICU nurses had the highest HAMs administration knowledge scores.

**Table 7:** Factors associated with nurses’ high-alert medications administrations knowledge score

Variable	Frequency (%) *N* = 280	Administration score Median [interquartile range]	Mean ± SD	*P*-value
Gender				
Male	153 (54.6)	7.0[5.0–8.0]	6.4 ± 2.0	0.002^a^
Female	127 (45.4)	6.0[4.0–7.0]	5.7 ± 1.9	
Age (year)				
< 25	37 (13.2)	6.0[5.0–7.0]	5.7 ± 1.9	0.022^b^
> 25–30	121(43.2)	6.0[4.0–7.0]	5.8 ± 2.1	
> 30–35	64 (22.9)	7.0[5.0–8.0]	6.5 ± 1.9	
> 35	58 (20.7)	7.0[5.0–8.0]	6.6 ± 1.9	
District				
Hebron	44 (15.7)	6.0[5.0–8.0]	6.3 ± 2.2	0.179^b^
Bethlehem	38 (13.6)	6.5[5.0–8.0]	6.5 ± 1.8	
Ramallah	58 (20.7)	6.0[5.0–8.0]	6.3 ± 2.2	
Nablus	74 (26.4)	6.0[4.8–7.0]	5.9 ± 1.8	
Tulkarm	26 (9.3)	5.0[4.0–7.0]	5.2 ± 1.9	
Jenin	40 (14.3)	6.0[4.3–8.0]	6.0 ± 2.2	
Education				
Diploma	96 (34.3)	6.0[4.3–7.0]	5.8 ± 2.1	0.243^b^
Bachelor	161 (57.5)	6.0[5.0–8.0]	6.2 ± 1.9	
Master	23 (8.2)	6.0[5.0–8.0]	6.4 ± 2.2	
Position				
Qualified Nurse ^c^	96 (34.3)	6.0[4.3–7.0]	6.9 ± 2.2	0.101^b^
Legal Nurse ^d^	170 (60.7)	6.0[5.0–8.0]	6.2 ± 1.9	
Head Nurse	14 (5.0)	7.5[5.0–9.0]	5.8 ± 2.1	
Wards				
ICU	71 (25.4)	7.0[5.0–8.0]	6.6 ± 2.0	0.011^b^
ER	62 (22.1)	6.0[4.8–8.0]	6.0 ± 2.2	
Pediatrics	75 (26.8)	5.0[5.0–7.0]	5.6 ± 1.9	
Medical	72 (25.7)	6.0[5.0–7.8]	6.1 ± 1.9	
Ward experience (year)				
< 2	95 (33.9)	6.0[5.0–7.0]	5.8 ± 1.9	0.301^b^
> 2–5	89 (31.8)	6.0[5.0–8.0]	6.0 ± 2.1	
> 5–1	62 (22.1)	7.0[5.0–8.0]	6.5 ± 1.9	
> 10	34 (12.1)	6.5[5.0–8.0]	6.3 ± 2.1	
Total experience (year)				
< 2	25 (8.9)	6.0[3.0–7.5]	5.2 ± 2.4	0.089^b^
> 2–5	69 (24.6)	6.0[5.0–7.0]	5.9 ± 1.8	
> 5–10	106 (37.9)	6.0[5.0–8.0]	6.1 ± 2.0	
> 10	80 (28.6)	7.0[5.0–8.0]	6.5 ± 2.0	
ICU training				
Yes	200 (71.4)	6.0[5.0–8.0]	6.2 ± 1.9	0.163^a^
No	80 (28.6)	6.0[4.3–8.0]	5.8 ± 2.2	
ER training				
Yes	234 (83.6)	6.0[5.0–8.0]	6.1 ± 2.0	0.528^a^
No	46 (16.4)	6.0[4.8–7.0]	6.0 ± 2.1	
HAMs training				
Yes	252 (90.0)	6.0[5.0–8.0]	6.2 ± 1.9	0.005^a^
No	28 (10.0)	5.0[4.0–6.0]	5.1 ± 2.1	
Training need				
Need	229 (81.8)	6.0[5.0–8.0]	6.0 ± 2.0	0.384^b^
No comment	33 (11.8)	7.0[5.0–8.0]	6.5 ± 2.1	
No need	18 (6.4)	7.0[4.8–8.0]	6.3 ± 1.6	

*ER* Emergency room, *ICU* Intensive care unit, *HAMs* High-alert medication

^a^Statistical significance of differences calculated using the Mann–Whitney U test

^b^Statistical significance of differences calculated using the Kruskal–Wallis test

^c^Qualified nurse with professional diploma degree in nursing

^d^Legal nurse with bachelor of science degree in nursing as the minimum educational requirement for professional nursing practice

### HAMs regulation knowledge score

The median HAMs regulation knowledge score was 6.0 (interquartile range: 5.0–7.0), and the mean score was 5.0 ± 1.7. As shown in Table 8, significant differences in HAMs regulation knowledge scores were found according to gender, ICU training, ER training and HAMs training (Mann-Whitney U test; *p*-value < 0.05), as well as educational level, hospital ward, district and nurse’s position (Kruskal-Wallis test; *p*-value < 0.05). Nurses who had ICU, ER and HAMs training had higher HAMs regulation knowledge scores than nurses who had not trained. Further, HAMs regulation knowledge score increased as educational level and nurse’s position increased; nurses with master degrees and head nurses achieved the highest HAMs regulation knowledge scores. Moreover, HAMs regulation knowledge scores were higher in nurses who were working in the ICU ward, compared to. HAMs regulation knowledge scores were also higher in male nurses compared to females, and nurses who were working in the Ramallah district compared to all other districts.

**Table 8:** Factors associated with nurses’ high alert medication regulation knowledge scores

Variable	Frequency (%) *N* = 280	Regulation score Median [interquartile range]	Mean ± SD	*P*-value
Gender				
Male	153 (54.6)	6.0[5.0–7.0]	6.1 ± 1.6	0.048^a^
Female	127 (45.4)	6.0[4.0–7.0]	5.7 ± 1.8	
Age (year)				
< 25	37 (13.2)	6.0[4.5–7.0]	5.7 ± 1.7	0.461^b^
> 25–30	121(43.2)	6.0[5.0–7.0]	5.9 ± 1.66	
> 30–35	64 (22.9)	6.5[5.0–7.0]	6.2 ± 1.6	
> 35	58 (20.7)	6.0[4.8–7.0]	5.7 ± 1.8	
District				
Hebron	44 (15.7)	6.0[5.0–7.0]	5.8 ± 1.7	0.024^b^
Bethlehem	38 (13.6)	5.0[4.0–7.0]	5.3 ± 1.6	
Ramallah	58 (20.7)	6.0[5.0–7.3]	6.4 ± 1.7	
Nablus	74 (26.4)	6.0[5.0–7.0]	6.1 ± 1.8	
Tulkarm	26 (9.3)	6.0[4.0–6.0]	5.2 ± 1.6	
Jenin	40 (14.3)	6.0[5.0–7.0]	5.9 ± 1.5	
Education				
Diploma	96 (34.3)	5.0[4.0–7.0]	5.28 ± 1.8	< 0.001^b^
Bachelor	161(57.5)	6.0[5.0–7.0]	6.2 ± 1.6	
Master	23 (8.2)	7.0[5.0–7.0]	6.3 ± 1.6	
Position				
Qualified Nurse ^c^	96 (34.3)	5.0[4.0–7.0]	5.3 ± 1.8	< 0.001^b^
Legal Nurse ^d^	170 (60.7)	6.0[5.0–7.0]	6.2 ± 1.6	
Head Nurse	14 (5.0)	7.0[6.0–7.0]	6.4 ± 1.2	
Wards				
ICU	71 (25.4)	7.0[6.0–7.0]	6.5 ± 1.6	0.013^b^
Emergency	62 (22.1)	6.0[5.0–7.0]	5.6 ± 1.6	
Pediatrics	75 (26.8)	6.0[4.0–7.0]	5.7 ± 1.8	
Medical	72 (25.7)	6.0[5.0–7.0]	5.8 ± 1.6	
Ward experience (year)				
< 2	95 (33.9)	6.0[5.0–7.0]	5.7 ± 1.7	0.248^b^
> 2–5	89 (31.8)	6.0[5.0–7.0]	5.8 ± 1.7	
> 5–10	62 (22.1)	6.0[5.0–7.0]	6.1 ± 1.7	
> 10	34 (12.1)	6.0[5.0–7.0]	6.2 ± 1.8	
Total experience (year)				
< 2	25 (8.9)	6.0[4.5–7.0]	5.7 ± 1.9	0.429^b^
> 2–5	69 (24.6)	6.0[4.0–7.0]	5.7 ± 1.7	
> 5–10	106 (37.9)	6.0[5.0–7.0]	6.1 ± 1.8	
> 10	80 (28.6)	6.0[5.0–7.0]	5.9 ± 1.6	
ICU training				
Yes	200 (71.4)	6.0[5.0–7.0]	6.1 ± 1.7	< 0.001^a^
No	80 (28.6)	5.0[4.0–6.0]	5.3 ± 1.5	
ER training				
Yes	234 (83.6)	6.0[5.0–7.0]	6.0 ± 1.7	0.012^a^
No	46 (16.4)	5.5[4.0–6.0]	5.3 ± 1.6	
HAM training				
Yes	252 (90.0)	6.0[5.0–7.0]	6.0 ± 1.7	0.014^a^
No	28 (10.0)	5.5[4.0–6.0]	5.1 ± 1.6	
Training need				
Need	229 (81.8)	6.0[5.0–7.0]	5.9 ± 1.7	0.782^b^
No comment	33 (11.8)	6.0[5.0–7.0]	5.7 ± 1.7	
No need	18 (6.4)	6.0[5.0–7.0]	5.9 ± 1.	

*ER* Emergency room, *ICU* Intensive care unit, *HAMs* High-alert medication

^a^Statistical significance of differences calculated using the Mann–Whitney U test

^b^Statistical significance of differences calculated using the Kruskal–Wallis test

^c^Qualified nurse with professional diploma degree in nursing

^d^Legal nurse with bachelor of science degree in nursing as the minimum educational requirement for professional nursing practice

### Relationship between administration and regulation knowledge of HAMs

The mean scores were 6.1 ± 2.0 for HAMs administration knowledge and 5.9 ± 1.7 for HAMs regulation knowledge, showing that nurses had higher administration knowledge than regulation knowledge. There was a significant positive correlation (r = 0.31, *p*-value < 0.001) between administration and regulation knowledge of HAMs.

### Factors associated with HAMs knowledge

As shown in Table 9, around two-thirds of respondents (188, 67.1%) had a score less than 70%, with only 32.9% of nurses achieving a score greater than or equal to 70%. This indicates poor knowledge regarding HAMs administration and regulation. Significant differences in knowledge scores were found according to gender, ward where the nurse currently works, HAMs and ICU training received, nurse’s position, and age (Chi-Square; p-value < 0.05). Male nurses and nurses with master and/or bachelor degrees achieved higher knowledge scores compared to those who did not. HAMs knowledge in paediatric nurses and nurses younger than 30 years was found to be poor. Furthermore, nurses who work in ICU, and had ICU and HAMs training, were found to be the most knowledgeable.

**Table 9:** Factors associated with knowledge of high-alert medications

Variable	Total frequency (%)	High score ≥ 70 *N* = 92	Low score < 70 *N* = 188	*P*-value
Gender				
Male	153(54.6)	62 (67.4)	91(48.4)	0.003
Female	127(45.4)	30 (32.6)	97 (51.6)	
Age (year)				
< 25	37 (13.2)	9 (9.8)	28 (14.9)	0.015
> 25–30	121(43.2)	31(33.7)	90 (47.9)	
> 30–35	64 (22.9)	30 (32.6)	34 (18.1)	
> 35	58 (20.7)	22 (23.9)	36 (19.1)	
District				
Hebron	44 (15.7)	14 (15.2)	30 (16.0)	0.052
Bethlehem	38 (13.6)	9 (9.8)	29 (15.4)	
Ramallah	58 (20.7)	27 (29.3)	31(16.5)	
Nablus	74 (26.4)	27 (29.3)	47 (25.0)	
Tulkarm	26 (9.3)	4 (4.3)	22 (11.7)	
Jenin	40 (14.3)	11(12)	29 (15.4)	
Education				
Diploma	96 (34.3)	23 (25)	73 (38.8)	0.072
Bachelor	161 (57.5)	60 (65.2)	101(53.7)	
Master	23 (8.2)	9 (9.8)	14 (7.4)	
Position				
Qualified Nurse^a^	96 (34.3)	23 (25)	73 (38.8)	0.042
Legal Nurse^b^	170 (60.7)	62 (67.4)	108 (57.4)	
Head Nurse	14 (5.0)	7 (7.6)	7 (3.7)	
Wards				
ICU	71(25.4)	36 (39.1)	35 (18.6)	0.002
ER	62 (22.1)	18 (19.6)	44 (23.4)	
Pediatrics	75 (26.8)	16 (17.4)	59 (31.4)	
Medical	72 (25.7)	22 (23.9)	50 (26.6)	
Ward experience (year)				
< 2	95 (33.9)	25 (27.2)	70 (37.2)	0.275
> 2–5	89 (31.8)	29 (31.5)	60 (31.9)	
> 5–10	62 (22.1)	24 (26.1)	38 (20.2)	
> 10	34 (12.1)	14 (15.2)	20 (10.6)	
Total experience (year)				
< 2	25 (8.9)	7 (7.6)	18 (9.6)	0.042
> 2–5	69 (24.6)	14 (15.2)	55 (29.3)	
> 5–10	106 (37.9)	38 (41.3)	68 (36.2)	
> 10	80 (28.6)	33 (35.9)	47 (25.0)	
ICU training				
Yes	200 (71.4)	74 (80.4)	126 (67.0)	0.02
No	80 (28.6)	18 (19.6)	62 (33.0)	
ER training				
Yes	234 (83.6)	79 (85.9)	155 (82.4)	0.468
No	46 (16.4)	13 (14.1)	33 (17.6)	
HAM Training				
Yes	252 (90.0)	88 (95.7)	164 (87.2)	0.027
No	28 (10.0)	4 (4.3)	24 (12.8)	
Training need				
Need	229 (81.8)	75 (81.5)	154 (81.9)	0.821
No comment	33 (11.8)	12 (13.0)	21(11.2)	
No need	18 (6.4)	5 (5.4)	13 (6.9)	

*ER* Emergency room, *ICU* Intensive care unit, *HAMs* High-alert medication

^a^Qualified nurse with professional diploma degree in nursing

^b^Legal nurse with bachelor of science degree in nursing as the minimum educational requirement for professional nursing practice

## Discussion

This is the first study of its kind in the Palestinian and Arab world. A review of the literature did not reveal any comprehensive studies of HAMs knowledge targeting nurses who practice anywhere in our country. Therefore, we need to evaluate nurses’ knowledge about HAMs in order to be able to provide appropriate education to update their knowledge, and also to prevent any improper handling of high risk medications that can put patients’ lives in danger. In addition, the knowledge gained from this study can be used for implantation of HAMs for the purpose of ensuring correct and safe administration, and to eliminate MEs that cause harm to patients [23]. The results of this study will establish the first baseline data regarding nurses’ HAMs knowledge, so that relevant educational and training programs can be initiated. Moreover, the findings of this study can help decision makers in academia to determine the proper courses to offer students in nursing schools, with respect to the use and handling of HAMs.

The response rate in our study was 93%; this is a very acceptable response rate, and is higher than a similar study in Taiwan [19], which had a response rate of 79.2%.

Several previous researchers [4, 10, 24, 25] have claimed that nurses have insufficient knowledge, and that this contributes to ME. This was the first study in Palestine to confirm this, especially with regard to HAMs; more than 60% (67.1%; 188/280) of respondents achieved HAMs knowledge scores less than 70%, with a mean score of 59.9 ± 15.1. Our results were consistent with a study in Taiwan [19], which revealed that nurses had insufficient knowledge about HAMs. Similarly, in China [26], researchers evaluated physicians’, pharmacists’, and nurses’ knowledge regarding HAMs, and found that nurses had the poorest knowledge.

Similar to previous studies, the current study found that the most common administration errors were related to lack of knowledge regarding IV drug administration, especially with regard to giving electrolytes as IV boluses (3% NaCl, 15% KCl, 10% Ca gluconate or 10% CaCl_2,_), and the dangerous consequences related to this. Electrolytes are commonly use and correct administration of them is vitally important. A Brazilian study [27] found that 74.0% of paediatric prescribed HAMs were for electrolytes. Further, in Taiwan [19], 30% of nurses were found to be giving electrolytes in an improper way.

It was surprising that 50.4% of nurses did not know that 500 mL of 3% NaCl should not be given as fast infusion for patients who have low sodium levels, as this may cause local pain and venous irritation. 3% NaCl is a concentrated solution and should be infused slowly with constant observation of sodium levels and oedema; IV administration of this solution should not exceed 100 mL\hour or 400 mL/24 h [28].

Moreover, 39.2% of nurses did not know that 10% CaCl_2_ should not be given as a fast IV push. CaCl_2_ injection should be administered slowly into a large vein, as it may lead to a cutaneous burning sensation and peripheral vasodilation. Additionally, we found that 31.4% of nurses did not know that 10% Ca gluconate and 10% CaCl_2_ are not interchangeable. These results confirmed that nurses had inadequate pharmacological knowledge about drugs they administer. The findings are consistent with those of Nadosi and Newall, who found that 74% of nurses in the north of England had insufficient pharmacology knowledge [10].

It is known that injection of KCl intravenously at excessively high speed may cause a cardiac arrest; this has been reported as a cause of death worldwide [27]. Reassuringly, 76.8% of nurses understood that 15% KCl should not be given as a fast IV push, though there was a small percentage of nurses who did not know this. Previous studies have recommended that nurses should not have free access to 15% KCl, and that this drug should not be stored in nursing units [19, 29, 30]. We recommend removing KCl from nursing units and re-educating nurses about its fatal effects and its proper administration.

Chemotherapy is considered one of the most dangerous cytotoxic drugs; errors in chemotherapy calculation can be catastrophic. Despite this, our study revealed that 53.6% of nurses did not understand that the adult chemotherapy calculation is based on BSA, whereas chemotherapy for children is based on BW. Similarly, this question had the lowest correct response rate (24.6%) in the study by Hsaio et al. [19]. A study in the USA suggested that it is important to increase nurses’ awareness about the fetal cytotoxic effect of chemotherapeutic agents, and the importance of accurate dosing of these agents [31].

Regarding Port-A route, 69.3% of nurses did not know about this route and its correct usage. As such, 45.7% of nurses reported using a Port-A route for blood withdrawal and drug injection generally, which is incorrect; this was the highest incorrect response rate among the questions assessing HAMs administration knowledge. This result contradicts the results of a Taiwanese study [19], where 64.9% of respondents answered this question correctly. Port-A route is not widely used in our hospitals, and most nurses did not know about it; this was the major cause of lack knowledge about it.

Insulin is one of the most important HAMs, as insulin overdose due to insufficient HAMs knowledge can lead to hypoglycemia. Using a dedicated syringe for the administration of insulin, and understanding that ‘unit’ is the dosage expression, not ‘cc’ or ‘mL’, are two necessary concepts for administration of insulin [19]. Despite that, 32.1% of nurses replaced an insulin syringe with a 1 mL syringe, and 52.9% replaced ‘unit’ with the abbreviation ‘U’. The use of ‘U’ instead of units might get confused with the number ‘0’, ‘11’ or with ‘cc’ [19]. These results concur with an Iranian study [32] which found that 40.5% of MEs occurred due to the use of acronyms and abbreviations. Another previous study [33] proposed that inadequate knowledge about diabetes contributes to insulin errors. In our study, 43.6% of nurses agreed with the presence of multiple drug concentrations to help them to choose. However, multiple concentrations and multiple dosing options can lead to error [34]. We suggest the use of dedicated insulin syringes, the encouragement of double checks, the separation of look-alike and sound-alike drugs, such as heparin and insulin, and the identification of the correct concepts related to insulin dosage expression, will reduce insulin-related administration errors.

Drug regulation is the control of drug use, administration, handling and storage. It is an important issue that needs attention and care from staff. In fact, drug regulation mistakes are responsible for a high percentage of risky medical errors, which is what motivated us to undertake this study. Regarding atracurium regulation, this drug should be refrigerated at 2–8 °C to preserve potency, and it should be used within 14 days, even if re-refrigerated [35]. However, 40.7% of respondents did not know that atracurium should be refrigerated, and they believed that it can be stored normally with other drugs. This drug is very dangerous as it may cause respiratory depression as a result of diaphragm muscle relaxation; the lack of knowledge on the correct handling of this drug is extremely concerning.

In our study, it was surprising that 47.9% of nurses used a spoon for paediatric dose expression. Spoons are available in various different sizes and are not sufficiently accurate to ensure a correct actual dose for children [36].

Fentanyl patch is a regular narcotic used for pain relief in patients with chronic pain; this patch is effective, well tolerated and has a long duration of action. Moreover, it has the advantage of avoiding IV administration. A previous report from the FDA described deaths and life-threatening adverse effects after health care professionals’ improperly recommended the fentanyl patch for the relief of headaches, or for after surgery, or for the treatment of pain associated with cancer and osteoarthritis, in patients who were not opioid tolerant [37]. Although there are studies describing the role of the fentanyl patch for the relief of chronic pain, only 48.6% of nurses knew about them. Whereas, in a previous study [19], this information was well known, and this question had the highest correct response rate of 91.1%. Fentanyl patch is not available in our hospitals and we suggest that it should be made available, given its advantages [38].

In order to find solutions for nurses’ insufficient knowledge about HAMs, it is important to know the most commonly encountered obstacles that lead them to make errors. Our results showed that the most common obstacle nurses encountered when administering HAMs was conflicting views between the nurse and doctor (37.9%). Moreover, 22.9% of respondents believed that confused prescription was another obstacle. Consistent with the results of another study [32], 78.5% of nurses believed that illegible prescriptions are responsible for the incidence of MEs. In order to resolve this problem, nurses, doctors and pharmacists should improve their communication skills. Further, establishment of a computerized system may reduce misunderstanding of prescriptions.

No established standard operating procedure for HAMs was the second most commonly reported obstacle (37.1% of nurses endorsed this).Several studies recommended that hospitals should develop their own list of HAMs, have a process for dealing with HAMs, and should establish this process in their hospital [39–41]. Therefore, we suggest establishing standard operating procedures for managing and handling HAMs.

Many studies have suggested that lack of knowledge is the main cause of MEs. For example, insufficient knowledge was the main obstacle that nurses encountered in Taiwan (75.4%) [19]. Similarly, inadequate knowledge of pharmacology (administration routes, drug side-effects, drug incompatibilities and the way of preventing them) was one of the human factors associated with MEs [32, 42]. The study of Johnson and Thomas [4] found that 77.1% of nurses agreed that it is important to update their knowledge of pharmacology. Further, 23.9% of nurses in our study considered insufficient knowledge a main obstacle, and 81.8% said that they needed additional training. In order to resolve this problem, HAMs training and teaching programs should be established in each hospital and nursing school. The findings of our study are consistent with the study of Hsaio et al. [19] which found that easy access to HAMs was the least common obstacle encountered during administration of HAMs.

Regarding factors associated with HAMs knowledge, we found significant differences according to gender, whereby males were more knowledgeable than females. Conversely, in Saudi Arabia [24], males were found to have the poorest pharmacology knowledge. All respondents in the Taiwanese study [19] were females. The current study found that HAMs knowledge score increased as the educational level and nurse’s position increased; nurses with a master degree and head nurses were the most knowledgeable groups. Conversely, in Saudi Arabia [24], head nurses were the least knowledgeable group. Further, in the study by Johnson and Thomas [4], there was no significant difference in the knowledge about medication according to level of education. In our study, no significant relationship between HAMs knowledge score and years of experience was found. This is consistent with the study of Johnson and Thomas [4], but inconsistent with the Taiwanese [19], as this study found that nurses’ knowledge increased as years of experience increased.

Nurses who had HAMs training and ICU training achieved higher knowledge score, which supports the importance of training and continuing education. Moreover, we found that nurses who were working in the ICU achieved higher knowledge scores. However, in a Jordanian study [43],there was no significant difference between ICU and other wards with regard to the rate of MEs. It is expected that nurses in the ICU are more knowledgeable about medications than nurses in other wards because they deal with more critical and acute cases; this was confirmed in our study.

As a strong point, the current study is, to our knowledge, the first study in the Palestine and Arab world assessing nurses’ knowledge of HAMs administration and regulation. In addition, the large sample size is strength of this study, which enabled good statistical power.

However, our study had some limitations. First, this study was of a comparative cross sectional design, whereby nurses were assessed only once at a specific period of time. Moreover, the generalizability of the results is limited as the sample was taken from government West Bank hospitals; private hospitals were not studied. Nonetheless, in Palestine, the government sector is considered the major provider of health care services.

## Conclusions

Our results provide evidence suggesting that nurses have insufficient knowledge of HAMs administration and regulation, especially with regard to the administration of IV boluses. Lack of knowledge was one of the obstacles nurses encountered during administration of HAMs; this can result in MEs. Nurses reported that they would like to have additional training to update their pharmacology knowledge. They also reported that conflicting views between nurses and doctors, and the lack of established standard operating procedures for HAMs, were the most commonly encountered obstacles during administration of HAMs; these contribute to the possibility of MEs. Males and nurses who were working in the ICU ward were the most knowledgeable. Moreover, nurses who had ICU and HAMs training achieved higher knowledge scores, which confirms the importance of HAMs training in improving nurses’ knowledge, and therefore, reducing MEs. We also found that as nurses’ educational level and position increased, so too did knowledge levels.

The findings of this study have a number of important implications for future practice including:

- We recommend that each hospital should establish its own list of HAMs, and this list should be updated regularly. Each hospital should also establish standard operating procedures for administration and regulation of HAMs.
- Additional continuing education and professional training regarding HAMs needs to be established in the teaching plans of nursing schools.
- Physicians, pharmacists and nurses need improved communication skills; this will help them to understand each other, ensuring clear communication between the relevant professionals, and thereby, reducing MEs.
- Continuous teaching and training programs about HAMs need to be established in each hospital, and clinical pharmacists should be involved in these programs, to provide valuable information about HAMs.
- Risk reduction strategies should be established for all steps of the medication use process, from preparation and storage to administration and monitoring; for example, look-alike drugs should be stored separately, and neuromuscular blocking agents should be stored in separate locked containers.

## Additional file

Additional file 1: Study questionnaire. This is the final English of the questionnaire that was used to obtain data that helps to assess the level of knowledge of high alert medications, the appropriate administration, and related error among nurses in West Bank, Palestine [1]. (DOCX 24 kb)

## Abbreviations

CaCl_2_: calcium chloride
HAMs: High Alert Medications
ICU: Intensive Care Unit
IV: Intravenous
KCl: potassium chloride
MEs: Medication Errors
NaCl: sodium chloride
SD: standard deviation

## References

[1] Bataille J, Prot-Labarthe S, Bourdon O, Joret P, Brion F, Hartmann JF (2015). High-alert medications in a French paediatric university hospital. J Eval Clin Pract.

[2] Wang HF, Jin JF, Feng XQ, Huang X, Zhu LL, Zhao XY, Zhou Q (2015). Quality improvements in decreasing medication administration errors made by nursing staff in an academic medical center hospital: a trend analysis during the journey to joint commission international accreditation and in the post-accreditation era. Ther Clin Risk Manag.

[3] Sheu SJ, Wei IL, Chen CH, Yu S, Tang FI (2009). Using snowball sampling method with nurses to understand medication administration errors. J Clin Nurs.

[4] Johnson J, Thomas M (2012). Medication errors: knowledge and attitude of nurses in Ajman. UAE Gulf Med J.

[5] Shamsuddin AF, Shafie SD (2012). Knowledge of nurses in the preparation and Administration of Intravenous Medications. Procedia - Social and Behavioral Sciences.

[6] Coyne E, Needham J, Rands H (2013). Enhancing student nurses' medication calculation knowledge; integrating theoretical knowledge into practice. Nurse Educ Today.

[7] Chen MJ, Yu S, Chen IJ, Wang KW, Lan YH, Tang FI (2014). Evaluation of nurses' knowledge and understanding of obstacles encountered when administering resuscitation medications. Nurse Educ Today.

[8] Simonsen BO, Daehlin GK, Johansson I, Farup PG (2014). Differences in medication knowledge and risk of errors between graduating nursing students and working registered nurses: comparative study. BMC Health Serv Res.

[9] Honey M, Lim AG (2008). Application of pharmacology knowledge in medication management by final year undergraduate nursing students. Contemp Nurse.

[10] Ndosi ME, Newell R (2009). Nurses' knowledge of pharmacology behind drugs they commonly administer. J Clin Nurs.

[11] Lo TF, Yu S, Chen IJ, Wang KW, Tang FI (2013). Faculties' and nurses' perspectives regarding knowledge of high-alert medications. Nurse Educ Today.

[12] El Sharif N, Samara I, Titi I, Awartani A (2011). Compliance with and knowledge about diabetes guidelines among physicians and nurses in Palestine. East Mediterr Health J.

[13] Shawahna R, Masri D, Al-Gharabeh R, Deek R, Al-Thayba L, Halaweh M (2016). Medication administration errors from a nursing viewpoint: a formal consensus of definition and scenarios using a Delphi technique. J Clin Nurs.

[14] Ayed A, Sayej S, Harazneh L, Fashafsheh I, Eqtait F (2015). The nurses' knowledge and attitudes towards the palliative care. J Educ Pract.

[15] Ahmead MK, Rahhal AA, Baker JA (2010). The attitudes of mental health professionals towards patients with mental illness in an inpatient setting in Palestine. Int J Ment Health Nurs.

[16] Zaid AN (2010). Attitude and perception of patients and health care practitioners toward oral sustained release dosage forms in Palestine. Saudi Pharm J.

[17] Ministry of Health, Palestinian Health Information Center (PHIC): Health Status, Palestine, 2014 [https://www.site.moh.ps/Content/Books/kD3bquHr7jbwK9f6VQJAsLDCuckgEDlCZUFa9ssb62m9Eim2le562D_ECDSNEboZRJwc6HyiggSMzKUPMeDJa2vkBNlAdZOGlvNuS9CHKJjGO.pdf].

[18] Raosoft Inc: Raosoft Sample Size Calculator [http://www.raosoft.com/samplesize.html].

[19] Hsaio GY, Chen IJ, Yu S, Wei IL, Fang YY, Tang FI (2010). Nurses’ knowledge of high-alert medications: instrument development and validation. J Adv Nurs.

[20] Wild D, Grove A, Martin M, Eremenco S, McElroy S, Verjee-Lorenz A, Erikson P (2005). ISPOR task force for translation and cultural adaptation: principles of good practice for the translation and cultural adaptation process for patient-reported outcomes (PRO) measures: report of the ISPOR task force for translation and cultural adaptation. Value Health.

[21] Chen HY, Boore JR (2010). Translation and back-translation in qualitative nursing research: methodological review. J Clin Nurs.

[22] Maneesriwongul W, Dixon JK (2004). Instrument translation process: a methods review. J Adv Nurs.

[23] Graham S, Clopp MP, Kostek NE, Crawford B (2008). Implementation of a high-alert medication program. Perm J.

[24] Asad M (2015). Assessment of nurses’ knowledge for possible occurrence of medication errors in Riyadh province, Saudi Arabia. Asian J Nurs Educ Res.

[25] Mrayyan MT, Shishani K, Al-Faouri I (2007). Rate, causes and reporting of medication errors in Jordan: nurses' perspectives. J Nurs Manag.

[26] Tang SF, Wang X, Zhang Y, Hou J, Ji L, Wang ML, Huang R (2015). Analysis of high alert medication knowledge of medical staff in Tianjin: a convenient sampling survey in China. J Huazhong Univ Sci Technolog Med Sci.

[27] Melo VV, Costa MS, Soares AQ (2014). Quality of prescription of high-alert medication and patient safety in pediatric emergency. Farm Hosp.

[28] Drugs.com: Calcium Chloride [http://www.drugs.com/pro/calcium-chloride.html].

[29] Lan YH, Wang KW, Yu S, Chen IJ, Wu HF, Tang FI (2014). Medication errors in pediatric nursing: assessment of nurses' knowledge and analysis of the consequences of errors. Nurse Educ Today.

[30] Lu MC, Yu S, Chen IJ, Wang KW, Wu HF, Tang FI (2013). Nurses' knowledge of high-alert medications: a randomized controlled trial. Nurse Educ Today.

[31] Schulmeister L (1999). Chemotherapy medication errors: descriptions, severity, and contributing factors. Oncol Nurs Forum.

[32] Cheragi MA, Manoocheri H, Mohammadnejad E, Ehsani SR (2013). Types and causes of medication errors from nurse's viewpoint. Iran J Nurs Midwifery Res.

[33] Heatlie JM (2003). Reducing insulin medication errors: evaluation of a quality improvement initiative. J Nurses Staff Dev.

[34] Health Research & Education Trust: Implementation guide to reducing harm from high-alert medications [http://www.ihconline.org/UserDocs/Pages/HRET_HEN_Change_Packages_AllMay2012.pdf].

[35] Drug.com: Atracurium besylate injection [http://www.drugs.com/pro/atracurium-besylate-injection.html].

[36] American Academy Of Pediatrics: AAP recommends using only metric dosing devices for children's medications not kitchen spoons [https://www.aap.org/en-us/about-the-aap/aap-press-room/pages/AAP-Recommends-Using-Only-Metric-Dosing-Devices-for-Children's-Medications-Not-Kitchen-Spoons.aspx].

[37] Food and Drug Administration (FDA): FDA 101 : Medication Errors [http://www.fda.gov/ForConsumers/ConsumerUpdates/ucm048644.htm].

[38] Ministry of Health: Palestinian essential drug list 2013 [http://pharmacy.moh.ps/Content/PDF/GXa4LIB4fU1MVPavE6Ceq6Ai_wijxbhTA93rak5SU4fpzuEjp.pdf].

[39] Institute for Safe Medication Practices: Your high-alert medication list—relatively useless without associated risk-reduction strategies [https://www.ismp.org/newsletters/acutecare/showarticle.aspx?id=45]. PMC5046995 27756996

[40] Grissinger M (2016). Your high-alert medication list is relatively useless without associated risk-reduction strategies. P T.

[41] Otero MJ, Moreno-Gomez AM, Santos-Ramos B, Agra Y (2014). Developing a list of high-alert medications for patients with chronic diseases. Eur J Intern Med.

[42] Shahrokhi A, Ebrahimpour F, Ghodousi A (2013). Factors effective on medication errors: a nursing view. J Res Pharm Pract.

[43] Mrayyan MT, Shishani K, Faouri I, Ammouri A (2008). Nurses’ perceptions of medication errors in Jordan. Jordan Med J.

