# Supplementary material for: Knowledge about the administration and regulation of high alert medications among nurses in Palestine: a cross-sectional study
Source: BMC Nurs. 2019 Mar 20;18:11. doi: 10.1186/s12912-019-0336-0 (PMC6425670; doi:10.1186/s12912-019-0336-0)
Supplement: Supplementary file 1 — Study questionnaire. This is the final English of the questionnaire that was used to obtain data that helps to assess the level of knowledge of high alert medications, the appropriate administration, and related error among nurses in West Bank, Palestine [1]. (DOCX 24 kb) [file 12912_2019_336_MOESM1_ESM.docx]

**Additional file 1: Study questionnaire. This is the final English of the questionnaire that was used to obtain data that helps to assess the level of knowledge of high alert medications, the appropriate administration, and related error among nurses in West Bank, Palestine [1].**

**Dear participate, the purpose of this questionnaire is to estimate nurses knowledge about High Alert Medications, the appropriate administration and related error, please answer the following questions, while insuring the confidently of the information provided**.

**The questionnaire including four sections:**

**First section:**

**1- Age: …………….**

**2- Gender: Male / Female**

**3- Place of residence: …………………**

**4- Collage / University name: ……………………**

**5- Graduation year: …………**

**6- Certificate degree: Diploma / Bachelor / Master**

**7- Hospital name: ………………**

**8- Hospital: Public / Private**

**9- Previous experiences in other hospitals: …………………..**

**10- Hospital department: ……………..**

**11-Years of experience in the department: ………………**

**12- Years of experience: …………………**

**13- Staff nurse / register nurse / intern nurse**

**14- Did you trained in ICU department: Yes / No**

**15- Dis you trained in Emergency department: Yes / No**

**16- Did you trained on handling High Alert Medications: Yes / No**

**Second section:**

**--The following questions is to estimate nurse’s knowledge of High Alert Medications administration:**

**Please answer the following question with yes or no:**

|  | **Yes** | **No** | **Don’t know** |
| --- | --- | --- | --- |
| **“Fast IV push 1:1000 epinephrine 1 ampule for patient who has mild allergic reaction”** |  |  |  |
| **“When an emergency happens, fast IV push 10% CaCL2 10 ml in 1-2 minutes”** |  |  |  |
| **“10% Ca gluconate and 10% CaCL2 are the same drug and interchangeable”** |  |  |  |
| **“‘cc’ or ‘ml’ is the dosage expression for insulin injection”** |  |  |  |
| **“For chemotherapy dose calculation, while adult based on BW, children BSA”** |  |  |  |
| **“When an emergency such as ventricular fibrillation happens, push fast 15% KCl 10 ml into IV”** |  |  |  |
| **“15%KCl better added to Ringer’s solution for rapid infusion”** |  |  |  |
| **“Insulin syringe can be replaced by 1ml syringe”** |  |  |  |
| **“Fast IV infusion of 3% NaCl 500 ml for patient who has low sodium level”** |  |  |  |
| **“Port-A route can be used for blood withdrawal and drug injection generally”** |  |  |  |

**Third section:**

**--The following questions is to estimate nurse’s knowledge of High Alert Medications regulation:**

**Please answer the following question with yes or no:**

|  | **Yes** | **No** | **Don’t know** |
| --- | --- | --- | --- |
| **“Use ‘amp’ or ‘vial‘ for dose expression instead of ‘mg‘ or ‘gm’”** |  |  |  |
| **“Use distinctive labeling on look-alike drugs”** |  |  |  |
| **“Use ‘U’ instead of unit for dose expression”** |  |  |  |
| **“For convenience, Heparin and Insulin should be stored together in the refrigerator”** |  |  |  |
| **“Each drug better have multiple concentrations for nurse to choice”** |  |  |  |
| **“If patient can tolerate, potassium can be administered orally instead of IV route”** |  |  |  |
| **“15% KCl is frequently used, so it should be easily and freely accessed by nurses”** |  |  |  |
| **“For pediatric dose, use teaspoon for dose expression”** |  |  |  |
| **“Taken Fentanyl skin patch as regulated narcotic”** |  |  |  |
| **“If a ward stores Atracurium for tracheal intubation, the drug should be stored with other drugs and easily access by nurses”** |  |  |  |

Adapted from [1] Hsaio GY, Chen IJ, Yu S, Wei IL, Fang YY, Tang FI: **Nurses' knowledge of high-alert medications: instrument development and validation**. *Journal of Advanced Nursing* 2010, **66**(1):177-190.

**Fourth section:**

**What are the obstacles you encounter during administer High Alert Medication (you can choose more than one answer):**

| **1. Insufficient knowledge.** |
| --- |
| **2. Have to accept oral order.** |
| **3. Confused prescription.** |
| **4. Inconsistent opinions between nurses.** |
| **5. Inconsistent opinions between doctor and nurse.** |
| **6. No reference for drug use.** |
| **7. Receive uncertain answers from colleagues.** |
| **8. Unclear dose calculation.** |
| **9. No established standard operating procedures for high alert medication.** |
| **10. No rigorous regulations for high alert medication.** |
| **11. Mix high alert medications with other drugs.** |
| **12. Easy access to high alert medications.** |
| **13. Find no suitable person to consult.** |
| **14. other** |

| **Please evaluate your Knowledge level:** |
| --- |
| **1. Sufficient.**  **2. Relatively sufficient.**  **3. Fair.**  **4. Insufficient.**  **5. Extremely insufficient.** |

| **Do you need more training:** |
| --- |
| **1. Yes, I need.**  **2. No comment.**  **3. No, I don’t need.** |

**Thank you for participate.**
